# Long-term outcomes after surgical dissection of inguinal lymph node metastasis from rectal or anal canal adenocarcinoma

**DOI:** 10.1186/s12885-019-5956-y

**Published:** 2019-07-24

**Authors:** Taro Tanabe, Dai Shida, Sho Komukai, Yuya Nakamura, Shunsuke Tsukamoto, Yukihide Kanemitsu

**Affiliations:** 1 Department of Colorectal Surgery, National Cancer Center Hospital, 5-1-1 Tsukiji, Chuo-ku, Tokyo, 1040045 Japan; 2 Division of Biomedical Statistics, Department of Integrated Medicine, Graduate School of Medicine, Osaka University, Osaka, Japan

**Keywords:** Inguinal lymph node metastasis, Rectal cancer, Anal canal cancer, Inguinal lymph node dissection, NCCN guidelines, TNM classification

## Abstract

**Background:**

The 8th edition of the tumor-node-metastasis (TNM) classification classifies inguinal lymph nodes as regional lymph nodes for anal canal carcinoma but non-regional lymph nodes for rectal carcinoma. This difference might reflect the different prognosis of inguinal lymph node metastasis from anal canal carcinoma and rectal carcinoma. However, long-term outcomes of inguinal lymph node metastasis from rectal or anal canal adenocarcinoma are unclear, which we aimed to investigate in this study.

**Methods:**

The study population included 31 consecutive patients with rectal or anal canal adenocarcinoma who underwent inguinal lymph node dissection with curative intent at the National Cancer Center Hospital from 1986 to 2017. Long-term outcomes were assessed and clinicopathologic variables analyzed for prognostic significance.

**Results:**

Of the 31 patients, 12 patients had rectal adenocarcinoma and 19 patients had anal canal adenocarcinoma. Synchronous metastasis were observed in 14 patients and metachronous metastasis in 17 patients. After dissection of inguinal lymph node metastasis with curative intent, the 5-year overall survival rate was 55.2%, with 12 patients surviving for more than 5 years. Median survival time was 66.6 months. Multivariate analyses revealed that location of primary tumor (rectum versus anal canal) was not a prognostic factor, whereas lateral lymph node metastasis and histological findings were independent prognostic factors.

**Conclusion:**

Given the good prognosis, inguinal lymph node metastasis in patients with rectal or anal canal adenocarcinoma appears to be regional rather than distant. If R0 resection can be achieved, inguinal lymph node dissection may be indicated for these patients.

## Background

Anal canal cancer is the most common type of gastrointestinal malignancy that metastasizes to inguinal lymph nodes (LNs). Whereas inguinal LN metastasis from anal canal cancer is classified as N2 in the 7th edition of the tumor-node-metastasis (TNM) classification when metastasis is unilateral, or as N3 when metastases are bilateral [1], it is categorized in the 8th edition as N1a (N1a: metastases in inguinal, mesorectal, and/or internal iliac nodes) [2, 3]. This classification was modified based on accumulating evidence from studies on anal canal squamous cell carcinoma [4, 5].

Most anal canal cancer cases in Western countries involve squamous cell carcinoma, which accounts for almost 90% of these cases [6]. In contrast, adenocarcinoma is the predominant histological subtype of malignancy arising in the anal canal in Asian countries such as Japan and China [7]. Specifically, adenocarcinomas account for 63% of anal canal cancers in China [8] and 74% in Japan [7], although anal canal cancer itself is a rare disease in these countries. Given the rarity of this disease, little is known about the long-term outcomes of inguinal LN metastasis from anal canal adenocarcinoma. The largest study cohort of patients (21 patients) with inguinal LN metastasis from anal canal adenocarcinoma to date was reported by Su et al., showing 5-year overall survival (OS) rate as 19.1% [8].

Inguinal LNs in rectal carcinoma are classified as non-regional LNs in the TNM classification [2]. Adenocarcinomas that originate from the lower rectum occasionally metastasize to inguinal LNs in a manner similar to anal canal cancer, with an incidence of approximately 2.0–4.5% [9, 10]. Some studies have reported that inguinal LN metastasis from rectal adenocarcinoma occurs as a consequence of locally advanced primary tumors or recurrent pelvic malignancy, and that in these cases, only systemic chemotherapy and radiotherapy should be considered due to the frequency of distant metastasis and poor prognosis [9, 11]. Other studies reported that solitary inguinal LN metastasis from rectal adenocarcinoma showed a favorable prognosis after LN excision and thus surgical treatment may be a reasonable therapeutic option for such patients [12, 13]. Accordingly, appropriate treatment strategies for inguinal LN metastasis from rectal adenocarcinoma are unclear, and surgical treatment for inguinal LN metastasis remains controversial.

The TNM 8th edition classifies inguinal LNs as regional LNs for anal canal carcinoma, but non-regional for rectal carcinoma [2]. No study to date has adequately accounted for this difference. Survival is thought to be an adequate indicator for determining regional versus distant metastasis. In this respect, this study aimed to investigate the long-term outcomes, specifically with respect to OS, of inguinal lymph node metastasis from rectal or anal canal adenocarcinoma, which makes it possible to speculate whether inguinal LN metastasis is regional or distant. In this study, because anorectal adenocarcinoma is sometimes difficult to determine its anatomical origin (rectum or anus) and thus rectal adenocarcinoma and anal adenocarcinoma sometimes overlap, and because treatment strategies for rectal adenocarcinoma and for anal adenocarcinoma are completely same according to the National Comprehensive Cancer Network (NCCN) guidelines 2018 [14, 15] (surgery, and sometimes followed by chemotherapy), and because of the limited sample size, inguinal LN metastasis from these two types was considered as a single entity and the combined data were analyzed.

## Methods

### Patients

Patients with inguinal LN metastasis from rectal or anal canal adenocarcinoma who underwent inguinal LN dissection with curative intent at the National Cancer Center Hospital from September 1986 to August 2017 were included in this study. Patients who had incomplete medical records and those who underwent only biopsy of inguinal LNs for diagnosis were excluded. Patients with inguinal LN metastasis from colon adenocarcinoma and patients with other histological types were also excluded.

The Institutional Review Board (IRB) of the National Cancer Center Hospital approved this retrospective study (IRB code: 2017–437).

### Anatomic definition of lower rectum and anal canal tumors

The TNM classification defines rectal carcinoma and anal canal carcinoma based on the anatomical location of the primary tumor. According to the TNM 8th edition [2], the anal canal begins where the rectum enters the puborectalis sling at the apex of the anal sphincter complex and ends with the squamous mucosa blending with the perianal skin. In the present study, tumor location was determined by colonoscopy and digital rectal examination before surgery. If the center of the tumor was located above the puborectalis sling, the tumor was defined as lower rectal cancer, and when below the puborectalis sling, as anal canal cancer.

### Treatment of rectal or anal canal adenocarcinoma in Japan

Preoperative treatment, including chemoradiotherapy and chemotherapy, prior to total mesorectal excision is the current standard for locally advanced rectal cancer in many Western countries [16]. However, in Japan, surgery with total mesorectal excision plus lateral lymph node dissection (LLND), without preoperative therapy is performed as the standard treatment for rectal cancer [17]. Thus, regardless of the clinical lateral lymph node status, LLND, including prophylactic dissection, without neoadjuvant chemoradiotherapy is usually performed for patients with locally advanced rectal or anal canal adenocarcinomas in Japan.

### Inguinal LN metastasis

All the patients in this study had clinically positive inguinal nodes detected on CT. In most cases, a biopsy of the inguinal LNs was not performed prior to inguinal node dissection. Patients with pathologically positive inguinal nodes were included in this study. Prophylactic inguinal LN dissection was not performed in cases of lower rectum adenocarcinoma or anal canal adenocarcinoma, without clinically positive inguinal nodes. Synchronous inguinal LN metastasis was defined as metastasis occurring within six months after the diagnosis of rectal or anal canal adenocarcinoma.

### Inguinal LN dissection

Technical details of inguinal LN dissection are described below. At 3 cm below the inguinal ligament, a slanting incision is made parallel to the inguinal ligament. Reaching above the femoral artery, a 6 cm incision is made along the femoral artery. Both superficial and deep inguinal LNs, including Cloquet’s nodes, are then dissected. After locating the femoral vein, the great saphenous vein is identified. All tissue between the fascia lata and Camper’s fascia within the standard template for inguinal node dissection is freed and the great saphenous vein is sacrificed. The inguinal ligament, adductor muscle, sartorius muscle, and the intersection point between these muscles surround the dissection area.

### Statistical analysis

The categorical variables were presented as frequencies, with percentages. Pearson’s chi-square test was used to compare categorical variables. The Kaplan-Meier method was used to estimate overall survival (OS) which was defined as the survival probability (in days) from the date of inguinal LN dissection to the date of death from all causes. The survival days were censored at May 1, 2018. We estimated OS for each covariate level, and we evaluated the association with each covariate using the logrank test. The results are shown as median survival and *p*-value. Multivariate Cox proportional hazards regression models with Firth’s modification [18], which were used to avoid sparse data bias and related problems, were subsequently fitted to evaluate the factors independently associated with OS. The prediction model was detected based on Akaike information criteria (AIC) from all conceivable models with different sets of covariates [19]. The results of the multivariate analyses were presented as hazard ratios (HRs), together with their 95% confidence interval (95% CIs) for the selected prediction model. A probability value of *P* < 0.05 was considered statistically significant. All statistical analyses were performed using the JMP13 software program (SAS Institute Japan Ltd., Tokyo, Japan) or R version 3.5.3 and the ‘coxphf’ package (R Project).

## Results

### Characteristics of the study cohort

One patient with an incomplete medical record, one patient who underwent a biopsy of an inguinal LN only for diagnostic purposes, and four patients with inguinal LN metastasis attributed to colon adenocarcinomas were excluded, leaving 19 patients with anal canal adenocarcinoma and 12 patients with lower rectal adenocarcinoma as the final study population. The patient characteristics and primary tumor information are summarized in Table 1. Of the 31 patients, 23 patients underwent abdominoperineal resection, six patients underwent pelvic exenteration, and two patients underwent intersphincteric resection for primary cancer. Thirty patients underwent surgery (total mesorectal excision plus LLND) without any preoperative therapy, and one patient received neoadjuvant chemoradiotherapy. LLND was not performed in eight (26%) patients for the following reasons: Five of these patients were clinical T1 stage, and the general condition of the other three patients was poor due to severe comorbidities or old age. Histological findings of the primary tumor showed a well- or moderately differentiated adenocarcinoma in 26 patients, poorly differentiated adenocarcinoma in three patients, and a mucinous adenocarcinoma in two patients. In all cases, the surgical margins were negative.

**Table 1:** General information on patients and primary tumor (*n* = 31)

Characteristic		Case (%)
Sex	Male	23 (74%)
	Female	8 (26%)
Age, years	< 65	15 (48%)
	≥65	16 (52%)
Surgical procedure	Abdominoperineal resection	23 (74%)
	Intersphincteric resection	2 (6%)
	Total pelvic exenteration	6 (20%)
Tumor size, cm	< 5	17 (55%)
	≥5	14 (45%)
Location	Rectum	12 (39%)
	Anal canal	19 (61%)
Depth of tumor invasion	pathological T1	3 (10%)
	pathological T2	7 (23%)
	pathological T3	11 (35%)
	pathological T4	10 (32%)
Mesorectal lymph node metastases	Yes	18 (58%)
	No	13 (42%)
Lateral lymph node metastases	Yes	12 (39%)
	No	11 (36%)
	LLND was not performed	8 (25%)
Distant metastases	M0	29 (93%)
	M1	2 (7%)
Histology	Well/moderately differentiated	23 (74%)
	Poorly differentiated/mucinous	8 (26%)
Pathological Stage	I	6 (19%)
	II	2 (7%)
	III	21 (67%)
	IV	2 (7%)

*LLND* lateral lymph node dissection

Clinicopathologic features of inguinal LN metastasis are described below. Median number of retrieved inguinal LNs was 7 (range, 1–22). We observed synchronous metastasis in 14 patients and metachronous metastasis in 17 patients. Two patients with synchronous inguinal LN metastasis also had liver metastasis. Bilateral inguinal LN metastasis was found in five patients. Seventeen patients had only one positive inguinal LN; four patients had two positive inguinal LNs; and 10 patients had more than two positive inguinal LNs. Five of 31 patients received adjuvant chemotherapy, and four patients received adjuvant radiotherapy, after inguinal LN dissection.

**Fig. 1:**
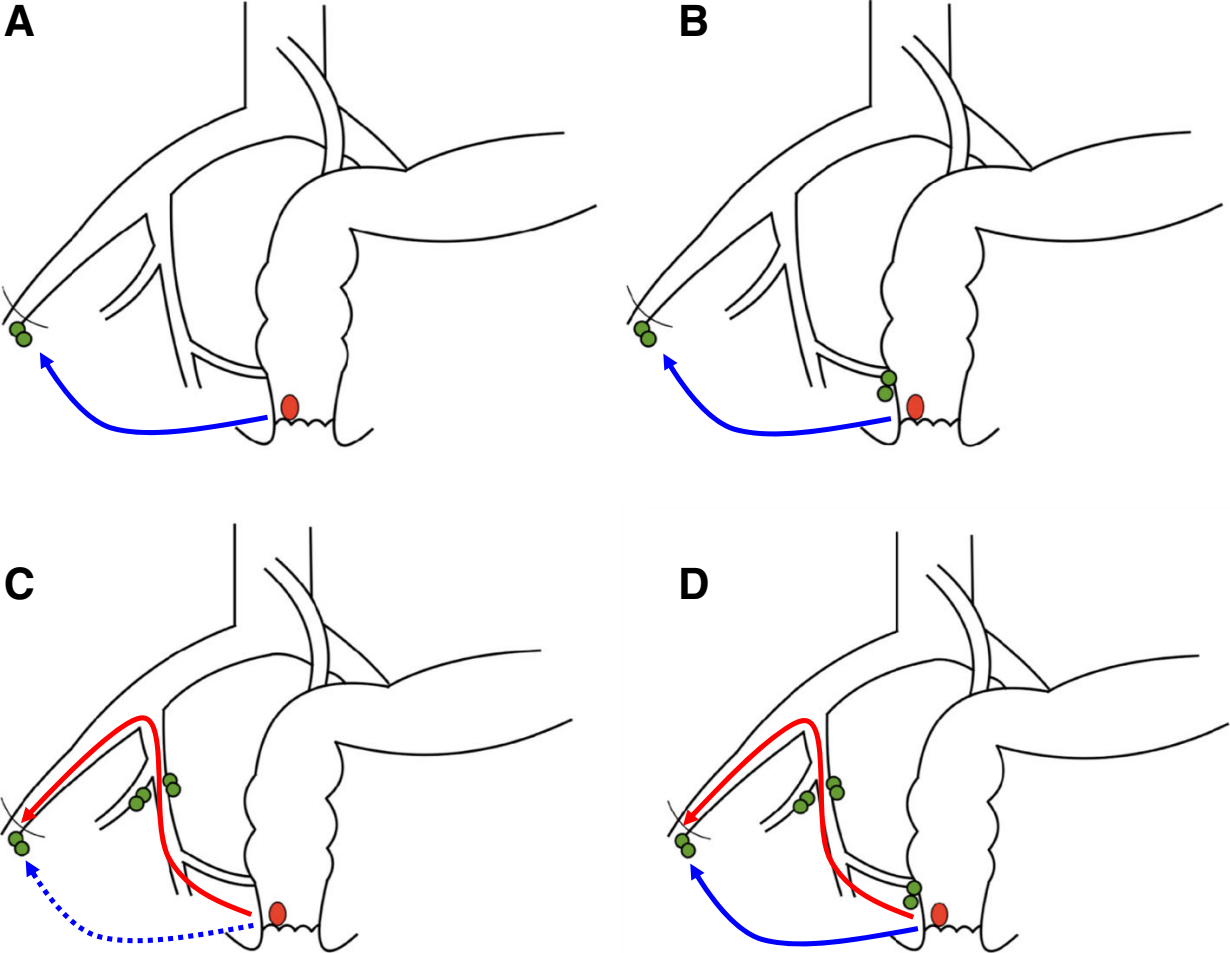
Types of lymph node (LN) metastasis from lower rectal and anal canal adenocarcinoma classified by the presence or absence of mesorectal LN metastasis and lateral LN metastasis. Arrows indicate speculative route of metastasis from tumor to ILNs. Blue arrow indicates direct route and red arrow indicates indirect route (through internal and external iliac vessels) to inguinal lymph nodes from the rectum or anal canal. **a** mesorectal LN(−), lateral LN(−): 10 cases; **b** mesorectal LN(+), lateral LN(−): 11 cases; **c** mesorectal LN(−), lateral LN(+): 3 cases; **d** mesorectal LN(+), lateral LN(+): 7 cases

Figure 1 shows types of inguinal LN metastasis classified by the presence or absence of mesorectal LN metastasis and lateral LN metastasis. Ten patients had neither mesorectal LN nor lateral LN metastasis (Fig. 1a), 11 patients had mesorectal LN metastasis without lateral LN metastasis (Fig. 1b), three patients had lateral LN metastasis without mesorectal LN metastasis (Fig. 1c), and seven patients had both mesorectal LN metastasis and lateral LN metastasis (Fig. 1d).

### Long-term outcomes after inguinal LN dissection

**Fig. 2:**
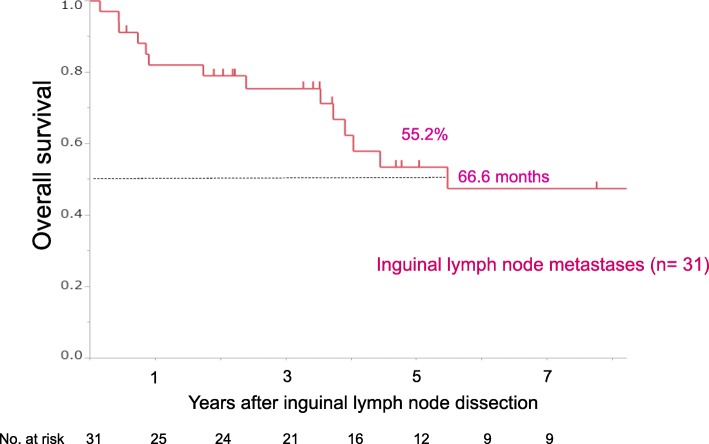
Overall survival curves for rectal and anal canal adenocarcinoma patients after inguinal LN dissection for inguinal lymph node metastasis (*n* = 31)

In the entire cohort, 3- and 5-year OS rates were 76.5 and 55.2%, respectively, with a median follow-up time for survivors of 47.5 months (range, 1.9–276.6 months). Median survival time (MST) was 66.6 months. Notably, 12 patients survived for more than five years (Fig. 2). No significant difference was found between prognosis of anal canal adenocarcinoma with inguinal LN metastasis and that of lower rectal adenocarcinoma with inguinal LN metastasis (*p* = 0.31).

Twenty-five patients experienced recurrence after inguinal LN dissection during the study period; 10 patients had local pelvic recurrence, four patients had inguinal LN recurrence (three on the other side, one on the same side), 11 patients had lung metastasis, three patients had liver metastasis, and one patient had peritoneal dissemination. As for timing of recurrence after inguinal LN dissection, 15 had recurrence within one year, and 10 had recurrence more than one year after inguinal LN dissection. Median relapse-free time was 10.3 months. Recurrence after inguinal LN dissection was treated by a multidisciplinary team approach including surgical resection (*n* = 4), radiotherapy (*n* = 4), chemotherapy (*n* = 14), and a combination of surgical resection and chemotherapy (*n* = 2).

### Factors affecting prognosis of inguinal LN metastasis

The univariate analyses revealed no significant association between the location of the primary tumor (rectum versus anal canal) and OS (*p* = 0.305). According to the multivariate analysis with Firth’s modification, a model composed of six covariates (see Table 2) was the optimal model based on the AIC from all conceivable models with different sets of covariates. Patients with lateral LN metastasis had a significantly worse prognosis (HR [95% CI]: 4.476 [1.42–15.28], *p* = 0.0011). Patients with histological findings of poorly differentiated or mucinous adenocarcinoma also had worse prognosis (HR [95% CI]: 7.995 [1.61–43.01], *p* = 0.012). In contrast, whereas tumor location, tumor size, presence of mesorectal LN metastasis, and timing of inguinal LN metastasis were selected in the model, these didn’t have significant HRs (Table 2). The other variables were not selected as the prognostic factor.

**Table 2:** Univariate and multivariate analyses of factors affecting survival in rectal or anal canal cancer patients with inguinal lymph node metastases

Variable	Category		Median overall survival (months)	Univariate analysis *p* value	Multivariate analysis		
					Hazard ratio	95% CI	*p* value
Sex	Male		54.1	0.160			
	Female		241.9				
Age, years	< 65		120.6	0.565			
	≥65		66.6				
Location of primary tumor	Rectum		120.6	0.305	Reference		
	Anal canal		54.1		2.01	0.65–7.12	0.230
Pathological T stage	T1, 2, 3		66.6	0.806			
	T4		121.7				
Tumor size, cm	< 5		49.1	0.745	Reference		
	≥5		121.7		0.51	0.16–1.59	0.247
Mesorectal lymph node metastases	No		120.1	0.409	Reference		
	Yes		54.1		2.06	0.56–7.08	0.262
Lateral lymph node metastases	No		241.9	0.025	Reference		
	Yes		45.3		4.48	1.42–15.28	0.011
Distant metastases	M0		120.6	0.811			
	M1		54.1				
Histology	Well/moderately differentiated		120.6	0.027	Reference		
	Poorly differentiated/mucinous		10.4		7.99	1.61–43.01	0.012
Number of positive inguinal LNs	< 3	(*n* = 21)	121.7	0.159			
	≥3	(*n* = 10)	51.5				
Site of inguinal LN metastases	Unilateral	(*n* = 26)	120.6	0.678			
	Bilateral	(*n* = 5)	51.6				
Timing of inguinal LN metastases	Synchronous	(*n* = 14)	49.1	0.236	Reference		
	Metachronous	(*n* = 17)	120.6		0.31	0.09–1.03	0.056

Data are presented as median or hazard ratio (95% CI)

*LN* lymph node

## Discussion

The present study found that, for long-term outcomes after inguinal LN dissection from rectal or anal canal adenocarcinoma with curative intent, MST was 66.6 months and 5-year OS was 55.2%. These results are noticeably better than previous data reported for inguinal LN metastasis from anal canal or rectal adenocarcinoma (MST, 8–14.8 months; 5-year OS, 0–19.1%) [8, 9, 11, 20]. This discrepancy could be due to the small sample size in the previous studies (8–32 patients) [8, 9, 11, 20], as well as recent developments in chemotherapy and the multidisciplinary team approach. In our study, although almost 80% of patients experienced recurrence after inguinal LN dissection, a multidisciplinary team approach that included surgical treatment and chemotherapy for recurrent tumors could have led to the better prognosis. Our MST of 66.6 months was also better than those reported for colorectal cancer patients with distant metastasis. According to the Analysis and Research in Cancers of the Digestive System database, MST was 19.3 months in colorectal cancer patients with liver metastasis, 24.6 months in those with lung metastasis, and 16.3 months in those with peritoneal metastasis [21]. Patients who underwent curative resection of liver metastasis, and thus are expected to have a favorable prognosis among stage 4 colorectal cancer patients, had 5-year survival rates of about 40% [22, 23], whereas patients who underwent curative resection of peritoneal metastasis had 5-year survival rates of about 30% [24, 25]. Thus, from the perspective of long-term outcomes, inguinal LN metastasis from rectal or anal canal adenocarcinoma appears to be regional rather than distant.

Previous studies have reported that, for rectal or anal canal adenocarcinoma with inguinal LN metastasis, unilateral inguinal LN metastasis, metachronous LN metastasis, and solitary inguinal LN metastasis are independent factors associated with a longer OS [12, 13, 20]. In contrast, we found that the absence of lateral LN metastasis and histological type of well or moderately differentiated adenocarcinoma were independent factors associated with longer OS in patients with inguinal LN metastasis from rectal or anal canal adenocarcinoma.

Lymph drainage at and proximal to the dentate line is directed toward the anorectal, perirectal, and paravertebral nodes and to some extent, the internal iliac system nodes, and lymph drainage below the dentate line mainly is directed to superficial inguinal LNs [26, 27]. There are two lymphatic routes from the rectum to inguinal LNs; one is a direct route [9], and the other is an indirect route which passes through internal and external iliac vessels. As shown in Fig. 2, we classified patterns of LN metastasis into four types based on the presence or absence of mesorectal LN and lateral LN metastasis. Figure 2a and b show inguinal LN metastasis via the direct route, and Fig. 2c and d by the indirect route (though lateral LNs), direct route, or both routes. Among the 10 patients with independent inguinal LN metastasis without metastasis to other areas, seven had a relatively small primary tumor (< 5 cm). In contrast, among the 14 patients with a tumor > 5 cm, 11 had mesorectal LN and/or lateral LN metastasis, indicating that large tumors invaded multiple lymphatic routes. According to multivariate analysis, patients with lateral LN metastasis had a poorer prognosis than patients without lateral LN metastasis. This suggests that inguinal LN metastasis by the indirect route leads to more progressive disease.

The TNM classification defines rectal carcinoma and anal canal carcinoma based only on the anatomical location of the primary tumor, without accounting for histological type. One issue with this is that both adenocarcinoma and squamous cell carcinoma, which originate from the anal canal, are classified in the same category despite their different treatment strategies. Namely, standard treatment for primary anal canal squamous cell carcinoma is chemoradiotherapy [4, 6], whereas that for adenocarcinoma is surgery. With regard to squamous cell carcinoma of the anal canal with inguinal LN metastasis, a previous study reported that the 5-year OS in patients with synchronous inguinal LN metastasis was 54.4%, and primary local control in the inguinal area after inguinal LN dissection was 68% [6]. In the present study of rectal and anal canal adenocarcinoma, 5-year OS was 55.2%, which is similar to that reported for anal canal squamous cell carcinoma patients.

This study has some limitations. First, since the study was a single-center retrospective analysis, biases may exist. Prospective studies will be needed to confirm our results. Second, the sample size was relatively small due to the rarity of inguinal LN metastasis from rectal or anal canal adenocarcinoma, although the number of patients who underwent inguinal dissection represented the largest sample reported to date. Third, treatment regimens varied among patients after inguinal LN dissection. Further prospective studies will be needed to confirm that inguinal LN metastasis from both rectal and anal canal adenocarcinoma is regional rather than distant.

## Conclusion

Based on the acceptable prognosis of patients who underwent inguinal LN dissection with curative intent, the presence of inguinal metastasis in patients with lower rectal and anal canal adenocarcinoma can be considered regional LN metastasis. If R0 resection can be achieved, inguinal LN dissection may be indicated in patients with inguinal LN metastasis from both rectal and anal canal adenocarcinoma.

## Data Availability

All data generated or analyzed during this study are included in this published article.

## Abbreviations

AIC: Akaike Information Criterion
AJCC: American Joint Committee on Cancer
CI: confidence interval
HR: hazard ratio
LLND: lateral lymph node dissection
LN: lymph node
NCCN: National Comprehensive Cancer Network
OS: Overall survival
TNM classification: Tumor-node-metastasis classification
